# Low platelet reactivity in patients with myocardial infarction treated with aspirin plus ticagrelor

**DOI:** 10.31744/einstein_journal/2022AO7001

**Published:** 2022-05-24

**Authors:** Thiago Guarato Rodrigues Costa, Marcelo Katz, Pedro Alves Lemos, João Carlos de Campos Guerra, Marcelo Franken, Antonio Eduardo Pereira Pesaro

**Affiliations:** 1 Hospital Israelita Albert Einstein São Paulo SP Brazil Hospital Israelita Albert Einstein, São Paulo, SP, Brazil.

**Keywords:** Myocardial infarction, Ticagrelor, Aspirin, Platelet aggregation, Hemorrhage, Platelet aggregation inhibitors

## Abstract

**Objective:**

Low platelet reactivity levels are associated with higher risk of bleeding in patients receiving dual antiplatelet therapy relative to patients with optimal platelet blockade. This study set out to evaluate the prevalence of low platelet reactivity in patients with acute myocardial infarction treated with ticagrelor and aspirin.

**Methods:**

Patients admitted with acute myocardial infarction who were already undergoing dual antiplatelet therapy with aspirin and ticagrelor were enrolled. Blood samples were collected 1 hour before and 2 hours after the maintenance dose of ticagrelor to investigate trough and the peak effects of the drug respectively. Platelet reactivity was measured by three methods: Multiplate^®^, PFA-100^®^ with Innovance^®^ PFA-P2Y cartridge and PFA-100^®^ with Collagen/ADP cartridge. Platelet reactivity was assessed in the presence of peak levels of ticagrelor and defined according to previously validated cut-offs for each method (<19 AUC, >299 seconds and >116 seconds respectively). The level of significance was set at p<0.05.

**Results:**

Fifty patients were enrolled (44% with ST-elevation). Median duration of DAPT was 3 days (interquartile range, 2-5 days). On average, peak and trough platelet reactivity were markedly low and did not differ between different methods. Low platelet reactivity was common, but varied according to analytic method (PFA-100^®^/Innovance^®^PFA-P2Y: 86%; Multiplate^®^: 74%; PFA-100^®^/Collagen/ADP: 48%; p<0.001).

**Conclusion:**

Low platelet reactivity was very common in patients with acute myocardial infarction submitted to dual antiplatelet therapy with ticagrelor and aspirin. Findings of this study justify the investigation of less intensive platelet inhibition strategies aimed at reducing the risk of bleeding in this population, such as lower dose regimens or monotherapy with P2Y_12_ inhibitors.

## INTRODUCTION

Dual antiplatelet therapy (DAPT) with aspirin and P2Y_12_ receptor antagonists is currently recommended for treatment of patients with acute coronary syndrome (ACS) worldwide.^(1)^ Several clinical trials have shown that DAPT with aspirin and ticagrelor or prasugrel is superior to DAPT with clopidogrel in reducing thrombotic and ischemic events, and is the current recommendation for patients with ACS.^(2,3)^ However, it is widely recognized that the protective effect of DAPT with newer P2Y_12_ antagonists against thrombosis is achieved at the expense of an increasing risk of hemorrhagic complications.^(4,5)^

Compared to older drugs, such as clopidogrel, the modern prasugrel and ticagrelor have been shown to have faster and more profound anti-platelet effects, with lower intra and interindividual variability.^(6–8)^ In patients undergoing DAPT with clopidogrel submitted to percutaneous coronary intervention (PCI), low platelet reactivity (LPR, *i.e*., severe platelet blockade) was associated with a two-fold higher risk of major bleedings, with no additional benefits in stent thrombosis reduction. In turn, patients with high platelet reactivity (*i.e.,* sub-optimal platelet inhibition) had a three-fold increase in the risk of stent thrombosis compared to patients within the so-called optimal platelet reactivity window.^(9)^ The introduction of more potent P2Y_12_ inhibitors in clinical practice stemmed from the fact that DAPT with aspirin may induce insufficient platelet inhibition and have limited clinical benefit. Patients undergoing DAPT with new P2Y_12_ antagonists have an increased risk of bleeding due to over-inhibition of platelet function.^(10)^ However, the anti-platelet effects of DAPT with new P2Y_12_ agents are poorly understood, especially in real-world treatment of patients with ACS.

## OBJECTIVE

To evaluate the therapeutic levels of dual antiplatelet therapy with ticagrelor and aspirin and to determine the frequency of over-inhibition of platelet function (*i.e.,* low platelet reactivity) in a real-world population of individuals with myocardial infarction treated with coronary intervention.

## METHODS

### Population and study design

This was a prospective, single-center observational study approved by the ethics committee of *Hospital Israelita Albert Einstein* (HIAE) (# 3.717.475, CAAE: 50157015.6.0000.0071), in compliance with the declaration of Helsinki. A signed informed consent was obtained from all patients. From March 2016 to August 2018, 50 individuals with acute myocardial infarction (MI) diagnosed in the emergency care unit within six hours of symptom initiation were consecutively enrolled. Inclusion criteria included diagnosis of MI according to international guidelines (elevation of serum myocardial necrosis markers, preferably troponin and/or CK-MB, associated with at least one of the following characteristics: ischemic symptoms, pathological Q-wave development on the electrocardiogram/ECG or ECG changes indicative of ischemia – *i.e.,* ST-segment elevation or depression, or imaging evidence of loss of viable myocardial area),^(11)^ treatment with a loading dose (180mg) of ticagrelor followed by the standard maintenance dose (90mg bid) combined with aspirin (100mg/day) and invasive coronary intervention. Therapeutic strategies and P2Y_12_ receptor inhibitors were selected by the medical team, which was unrelated to this study. Exclusion criteria were as follows: previous use of oral anticoagulants, active bleeding, previous hematologic disorder, use of thrombolytic agents, active infection, cardiogenic shock, renal failure requiring dialysis, use of non-ticagrelor P2Y_12_ and/or GPIIbIIIA inhibitors.

### Laboratory methods

Platelet function tests of the 50 patients included in this study were carried out using multiple electrode aggregometry (MEA) ADP (adenosine diphosphate)-induced (Multiplate^®^ analyzer, Roche, Germany) and the PFA-100^®^ system (Siemens, Germany) with Innovance^®^ PFA^®^P2Y and PFA^®^Collagen/ADP cartridges used according to manufacturer instructions. Tests were performed using quality laboratory practices.

To assess platelet reactivity at 2 time points (highest and lowest bioavailability), whole blood samples were collected by venipuncture 1 hour before and 2 hours after (trough and peak level respectively) the morning maintenance dose of ticagrelor (90mg). Samples were immediately sent to the clinical laboratory and processed within 2 hours of collection, in compliance with platelet stability times in whole blood.^(12)^ Tests results were not shared with care teams are were only seen by the research team. Samples were collected by venipuncture at two sites, preferably on different limbs, in order to eliminate potential interferences with platelet activity associated with prior venipuncture or catheter insertion. Blood was drawn into Hirudin anticoagulant tubes and in 3.2% citrated tubes for platelet aggregation tests (Multiplate^®^ analyzer and PFA-100^®^ system respectively). Patients were only followed while hospitalized. Metabolic and hematological laboratory test results were obtained from medical records.

Multiplate^®^ analyzer assays were processed using the ADPtest^®^ agonist, which promotes platelet activation via ADP platelet receptors and is sensitive to P2Y_12_ receptor inhibitors, using 6.5*µ*M as the final ADP concentration. Low platelet reactivity was defined as platelet reactivity less than 19 AUC in the Multiplate^®^ assay. This cut-off was recommended by two international expert consensus statements^(13,14)^ and supported by data from a collaborative analysis of more than 20,000 patients treated with DAPT and coronary angioplasty.^(9)^

For the PFA-100^®^ system, samples were processed using a hematology analyzer for quality assessment (testing is highly influenced by hematocrit level and platelet count). Approved samples were inserted into cartridges without blistering. Platelet function analysis is performed automatically on a closed platform and assessed by occlusion time. There is no consensus regarding therapeutic range or bleeding risk threshold for PFA-100^®^ test results for both cartridges. According to Breet et al.^(15)^ only patients with occlusion time longer than 299 seconds (PFA^®^P2Y) or 116 seconds (Collagen/ADP cartridge) are defined as LPR patients.

### Statistical analysis

Continuous variables were expressed as mean±standard deviation (SD) or medians and interquartile ranges, as appropriate. Categorical variables were described as absolute and relative frequencies. The Mann-Whitney test and the paired Student’s *t*-test were used to compare continuous variables between groups. Trough and peak of platelet reactivity measured using different methods were compared using the McNemar test. Baseline clinical parameters were compared between normal and LPR patients using the χ^2^, the Fisher’s or a likelihood ratio test. All statistical tests were bilateral. The level of significance was set at p<0.05. Statistical analyses were performed using IBM-SPSS for Windows, version 22.0.

## RESULTS

A total of 50 patients were enrolled (84% of males, age 59.3±12.7 years, 28% with diabetes and 44% with ST-elevation MI at initial assessment). All patients were submitted to coronary angioplasty and received drug eluting stents. The median time from ticagrelor therapy initiation to blood collection was 3 days (interquartile range, 2-5 days). Clinical characteristics of patients with and without LPR at peak drug activity, as measured by Multiplate^®^, are shown in table 1.

**Table 1:** Clinical characteristics of patients with and without low platelet reactivity, as measured by Multiplate^®^ within 2h of ticagrelor maintenance dose (peak)

Characteristics		Patients without LPR (n=13)	Patients with LPR (n=37)	p value
Male sex, n (%)		10 (76.9)	32 (86.5)	0.413
Age, years (mean±SD)		64.8±14.2	57.4±11.8	0.071
BMI (mean±SD)		28.1±3.18	28.5±3.61	0.727
Risk factors for CV, n (%)				
	Hypertension	7 (53.8)	19 (51.4)	0.877
	*Diabetes mellitus*	3 (23.1)	11 (29.7)	0.734
	Dyslipidemia	7 (53.8)	15 (40.5)	0.406
MI type, n (%)				
	STEMI	5 (38.5)	17 (45.9)	0.640
	Previous MI	2 (15.4)	10 (27.0)	0.480
Coronary angioplasty, n (%)				
	Anterior descending artery	9 (69.2)	23 (62.2)	0.347
	Right coronary artery	4 (30.8)	16 (43.2)	0.777
	Circumflex coronary artery	7 (53.8)	7 (18.9)	0.206
Medications, n (%)				
	ACE inhibitors	4 (30.8)	17 (45.9)	0.340
	Calcium-channel blockers	3 (23.1)	2 (5.4)	0.103
	Beta-blockers	8 (61.5)	20 (54.1)	0.640
	Aspirin	13 (100)	37 (100)	>0.999
	LMWH	9 (69.2)	21 (56.8)	0.430
	Oral antidiabetics	3 (23.1)	11 (29.7)	0.734
	Statins	13 (100)	34 (91.9)	0.558
Laboratory tests, mean±SD				
	Hemoglobin, g/dL	13.6±1.6	14.3±1.3	0.116
	Platelets, x10^3/mL	220.3±66.0	205.6±57.0	0.448
	Creatinine, mg/dL	1.1±0.5	1.1±0.7	0.987
	Glucose, mg/dL	147.4±74.7	128.3±43.0	0.270
	Total cholesterol, mg/dL	188.0±62.0	137.2±40.5	0.009*
	HDL cholesterol, mg/dL	40.7±17.4	38.1±7.5	0.676
	LDL cholesterol, mg/dL	120.3±57.3	74.0±36.7	0.009*
	Triglycerides, mg/dL	137.2±64.4	137.2±60.7	0.990
	Troponin, pg/mL (median [min;max])	7.650 [109;152.000]	18.000 [77.5;179.000]	0.877
	LVEF	0.5±0.1	0.6±0.1	0.088

* Comparison between patients (Pts) with and without LPR; p<0.05 indicates significant differences between groups.

BMI: body mass index; CV: cardiovascular; MI: myocardial infarction; ACE inhibitors: Angiotensin converting enzyme inhibitors; LMWH: low molecular weight heparin; LVEF: left ventricular ejection fraction; LPR: low platelet reactivity; SD: standard deviation; min-max: minimum-maximum.

**Figure 1:**
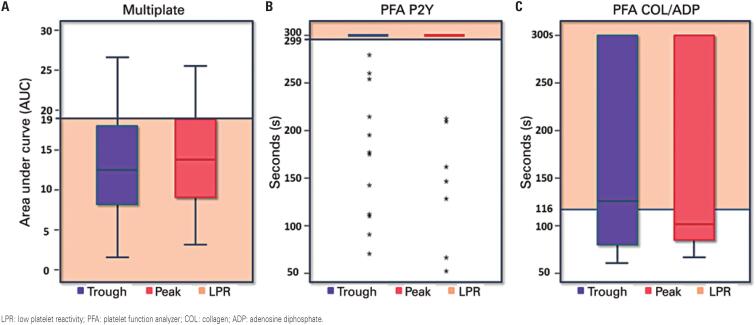
Boxplot of trough and peak blood levels of ticagrelor measured by different methods. (A) Multiplate^®^ ADP tests (AUC units); (B) PFA^®^ Col/ADP (seconds); (C) PFA^®^ P2Y (seconds). Continuous lines indicate cut-off values for low platelet reactivity

Peak and trough platelet reactivity were markedly low and did not differ significantly when measured using Multiplate^®^ (mean±SD, 14±6 AUC and 13±7 AUC respectively; p=0.101), PFA^®^P2Y (median [min;max], 300s [71; 300] and 300s [53,300] respectively; p=0.286) or PFA^®^Collagen/ADP (median [min;max], 101s [64,300] and 125s [59,300] respectively; p=0.347) (Figure 1). The prevalence of LPR was also similar at peak and trough of the drug, as measured by the three methods (74% and 82%, p=0.09; 86% and 76%, p=0.58 and 48% and 52%, p=0.71; Multiplate^®^, PFA^®^P2Y and PFA^®^ Collagen/ADP respectively).

**Figure 2:**
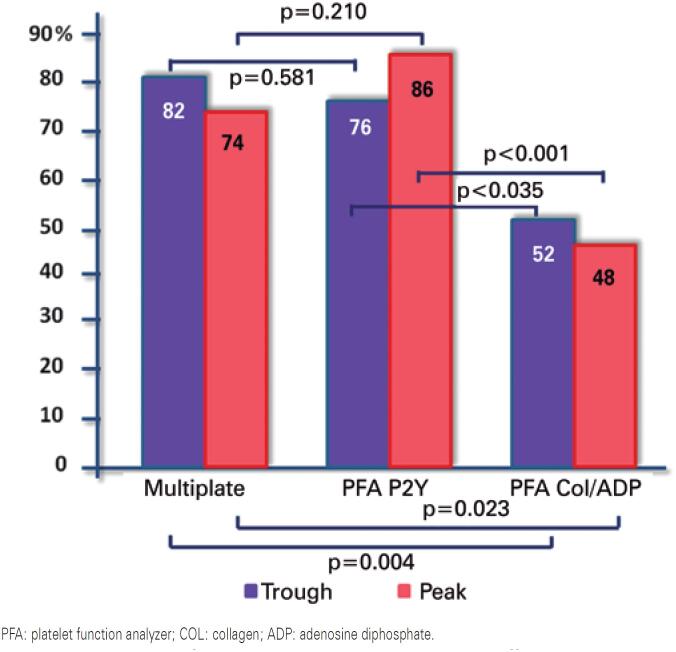
Prevalence of low platelet reactivity detected by different methods. P values are shown for comparative analysis of the prevalence of low platelet reactivity across different methods

Comparative analysis of results obtained by methods revealed that the prevalence of LPR during peak ticagrelor activity was higher when measurements were made using Multiplate^®^ and PFA^®^P2Y relative to PFA^®^ Collagen/ADP (74%, 86% and 48% respectively; p<0.001) (Figure 2).

## DISCUSSION

The main finding of this study was that patients with MI undergoing DAPT with ticagrelor and aspirin have a high prevalence of LPR and are therefore at a high risk of bleeding, with no theoretical gain in terms of protection from ischemic events.

Prior studies have shown that platelet reactivity can be used to stratify the risk of thrombotic and/or hemorrhagic events in ACS patients receiving with DAPT.^(16)^ Bleeding complications are strongly associated with death in ACS patients and the risk of bleeding during treatment with antiplatelet drugs may be higher in more vulnerable subgroups (*i.e.,* elderly individuals and patients with low body weight, renal failure, etc.).^(17,18)^ A collaborative analysis of 17 studies with 20,839 patients submitted to DAPT and coronary angioplasty (97% treated with clopidogrel and 3% with prasugrel) revealed that 20% of patients had LPR (*i.e.,* Multiplate^®^ values <19 AUC). Importantly, when compared to patients with optimal platelet reactivity (*i.e*., Multiplate^®^ values between 19 and 46 AUC), this subgroup had a two-fold higher in-hospital PCI-related risk of bleeding (risk ratio: 1.74, 95%CI: 1.47-2.06, p<0.00001) without further reduction of ischemic events.^(9)^

In this study, the prevalence of LPR measured using the Multiplate^®^ or the PFA^®^P2Y method was similar. However, when measured using PFA^®^ Collagen/ADP, the prevalence was approximately 30% lower. Importantly, different from prior studies,^(7,8)^ this analysis included a contemporary approach to evaluate P2Y receptor inhibition by ticagrelor (Multiplate^®^) and adopted an evidence-based cut-off for hemorrhagic adverse event prediction in patients submitted to coronary angioplasty.^(13)^

The prevalence of LPR in MI patients treated with ticagrelor and aspirin in this study are in keeping with findings of other small trials using different laboratory methods in patients with acute or stable coronary disease. In a substudy of the PLATO trial, which investigated platelet responses in MI patients treated with clopidogrel or ticagrelor combined with aspirin, platelet reactivity was lower in the ticagrelor arm, regardless of measurement method (PRP [ADP 20*µ*M and ADP 5*µ*M]; vasodilator-stimulated phosphoprotein (VASP) and VerifyNow).^(7)^ Of note, mean values of platelet reactivity measured using the VerifyNow assay in patients treated with ticagrelor (peak drug activity) were approximately 31 PRU. Such values are far lower than the cut-off value (<85 PRU) that was subsequently recommended by two international expert consensus statements.^(13,14)^ Moreover, in a PEGASUS-TIMI 54 substudy involving stable coronary artery disease patients with history of MI, platelet reactivity was tested after administration of different doses of ticagrelor (60mg and 90mg) and compared with placebo. In that study, patients also received aspirin. Patients treated with ticagrelor had very low levels of platelet reactivity (from 20±19 to 29±39 PRU) regardless of dose.^(8)^

Interestingly, in the THEMIS trial, which examined whether combining ticagrelor and aspirin might improve ischemic outcomes in patients with stable coronary artery disease and diabetes, the dose of ticagrelor was decreased during the trial (from 90mg to 60mg bid). Patients treated with ticagrelor had a lower incidence of ischemic cardiovascular events. However, the risk of major bleeding was significantly (2.3 fold) higher in these patients relative to those treated with placebo.^(19)^

From a clinical perspective, evidence from randomized and observational studies comparing ticagrelor with other P2Y_12_ receptor inhibitors suggests DAPT with ticagrelor may carry an increased risk of bleeding. In a meta-analysis comparing ticagrelor and prasugrel, prasugrel was associated with lower myocardial infarction rates (odds ratio - OR=0.54; 95%CI: 0.29-0.99; p=0.05) and significantly lower bleeding rates (OR=0.75; 95%CI: 0.59-0.95; p=0.02), in spite of similar impacts of both strategies on other ischemic outcomes and mortality.^(20)^ In an Swedish observational study comparing clopidogrel and ticagrelor use in 45,206 cardiac patients, ticagrelor was associated with a lower risk of combined ischemic outcomes (death, myocardial infarction or stroke). However, the rate of in-hospital PCI-related bleeding events was significantly higher (3.7% *versus* 2.7%; hazard ratio=1.57; 95%CI: 1.30-1.90).^(4)^

Therefore, in keeping up with existing evidence, this study demonstrated a confirmed marked platelet inhibition by DAPT with ticagrelor and aspirin in MI patients. Findings also revealed similar LPR levels before and after the morning dose of ticagrelor, suggesting a significant residual inhibitory effect even at trough blood concentrations. High levels of platelet aggregation inhibition may be explained by the direct effect and potent reduction of thromboxane A2 and other platelet agonists by ticagrelor combined with the irreversible antiplatelet effect of acetylsalicylic acid (ASA).^(21)^ Results presented beg the question of whether less intensive platelet inhibition strategies based on modern P2Y_12_ receptor antagonists might attenuate LPR and possibly reduce the risk of bleeding without increasing thrombotic events in this clinical setting. For instance, findings of this study may inform future clinical trials testing monotherapy with ticagrelor (*i.e*., without aspirin) in patients submitted to coronary interventions.

Importantly, a number of recent trials have compared the effects of DAPT with monotherapy with ticagrelor in ACS. As an example, two clinical trials compared ticagrelor monotherapy after short-term DAPT with conventional DAPT after PCI. Patients submitted to monotherapy with ticagrelor had a lower risk of major bleeding, with no evidence of increased ischemic risk relative to patients receiving conventional DAPT.^(22,23)^ Combined with current evidence, these findings suggest monotherapy with P2Y_12_ inhibitors following short-term DAPT is safe and may reduce the risk of bleeding, regardless of patient age.^(24,25)^ A recent small trial with aspirin-free prasugrel monotherapy immediately after angiography and coronary stent implantation in stable patients also suggested this strategy is safe and not conducive to stent thrombosis.^(26)^

This study has important limitations. It is a single center study with a small sample of patients. Therefore, clinical characteristics associated with LPR may have been underestimated. One example is the numerical difference in age between patients with and without LPR, which lacked statistical significance. A larger cohort of patients might have settled this matter. Two platelet function tests based on different methods and with different particularities were used to detect responsiveness to ADP receptor inhibitors. Results obtained with different methods are expressed in units of measurement that cannot be numerically compared. However, all tests indicated a high rate of LPR in the target population. Cut-off values used to define LPR measured by PFA methods are not supported by clinical evidence regarding bleeding prediction in patients with coronary artery disease. Nevertheless, high levels of LPR were detected not only by PFA, but also by Multiplate testing, according to cut-off values recommended by international expert consensus and supported by robust clinical data in patients submitted to coronary interventions.^(13,14)^ Although current data are derived primarily from trials with clopidogrel and aspirin combination therapy, the existing body of evidence and the expert consensus suggest potent P2Y_12_ receptor inhibition is associated with an increased risk of bleeding in patients submitted to DAPT. Importantly, this piece of information may not apply to alternative antiplatelet strategies implemented after coronary interventions, such as monotherapy with ticagrelor, for which different cut-off values for outcome prediction remain to be determined.

## CONCLUSION

In conclusion, patients with acute myocardial infarction submitted to invasive management and therapy with ticagrelor and aspirin had a high prevalence of low platelet reactivity. Findings of this study justify future clinical trials testing less intensive platelet inhibition strategies with potentially lower bleeding risk in this clinical setting, such as low dose regimens or monotherapy with ticagrelor.
